# When Low Back Pain Unveils an Unusual Diagnosis: Persistence of Gallstones After Cholecystectomy

**DOI:** 10.7759/cureus.80454

**Published:** 2025-03-12

**Authors:** Ana Lopes Gomes, Ana Durães, Tânia Gomes, Ana Rita Gonçalves

**Affiliations:** 1 Family Medicine, Unidade de Saúde Familiar (USF) São Vítor, Unidade Local de Saúde de Braga (ULS Braga), Braga, PRT

**Keywords:** biliary stone, cholecistectomy, litiasic drenage, low-back pain (lbp), traumatic hematoma

## Abstract

Persistence of biliary stones after cholecystectomy is a rare condition that requires early diagnosis and timely intervention to prevent complications. We present the case of a 67-year-old female with a history of hypertension, dyslipidemia, and cholecystectomy (2009), who developed ectopic lithiasic drainage through a fistulous tract following a traumatic hematoma in the dorsolumbar region.

The patient experienced inflammatory signs and spontaneous drainage of stones, later confirmed as calcium oxalate. Imaging and endoscopic procedures revealed persistent biliary stones and a fistulous cavity in the hepatorenal space, leading to multiple episodes of stone expulsion and abscess drainage. Surgical intervention, including partial fistulectomy and debridement with extraction of biliary stones, resulted in symptom resolution.

This case underscores a rare post-cholecystectomy complication and highlights the importance of an integrated multidisciplinary approach involving primary and secondary care. Early intervention and longitudinal follow-up by the family physician were critical in achieving a favorable outcome.

## Introduction

Cholelithiasis affects approximately 20% of the adult population [1], and for symptomatic cases, laparoscopic cholecystectomy has become the preferred treatment modality in recent decades. However, laparoscopic cholecystectomy can lead to certain complications. Dropped gallstones occur in approximately 7% (but as many as 30%) of laparoscopic cholecystectomies as a result of gallbladder perforation during surgical dissection and extraction [2-4]. Most of these stones are evacuated intraoperatively, but fragmented and inaccessible stones are left in the peritoneal cavity in an estimated 2.4% of laparoscopic cholecystectomies [3]. In most cases, these stones cause no complications. In rare instances, dropped gallstones become symptomatic, resulting in a wide range of complications with considerable associated morbidity.

Understanding these potential complications, such as infection or sepsis, is clinically relevant and underscores the importance of this issue. Diagnosing this condition can be challenging due to unusual clinical presentations, but early diagnosis, using imaging techniques and laboratory parameters, is crucial for timely treatment and prevention of these complications.

## Case presentation

A 67-year-old female with a personal history of hypertension and dyslipidemia. As a previous surgical history, she underwent laparoscopic cholecystectomy in 2009 and appendectomy in 2000.

**Figure 1:**
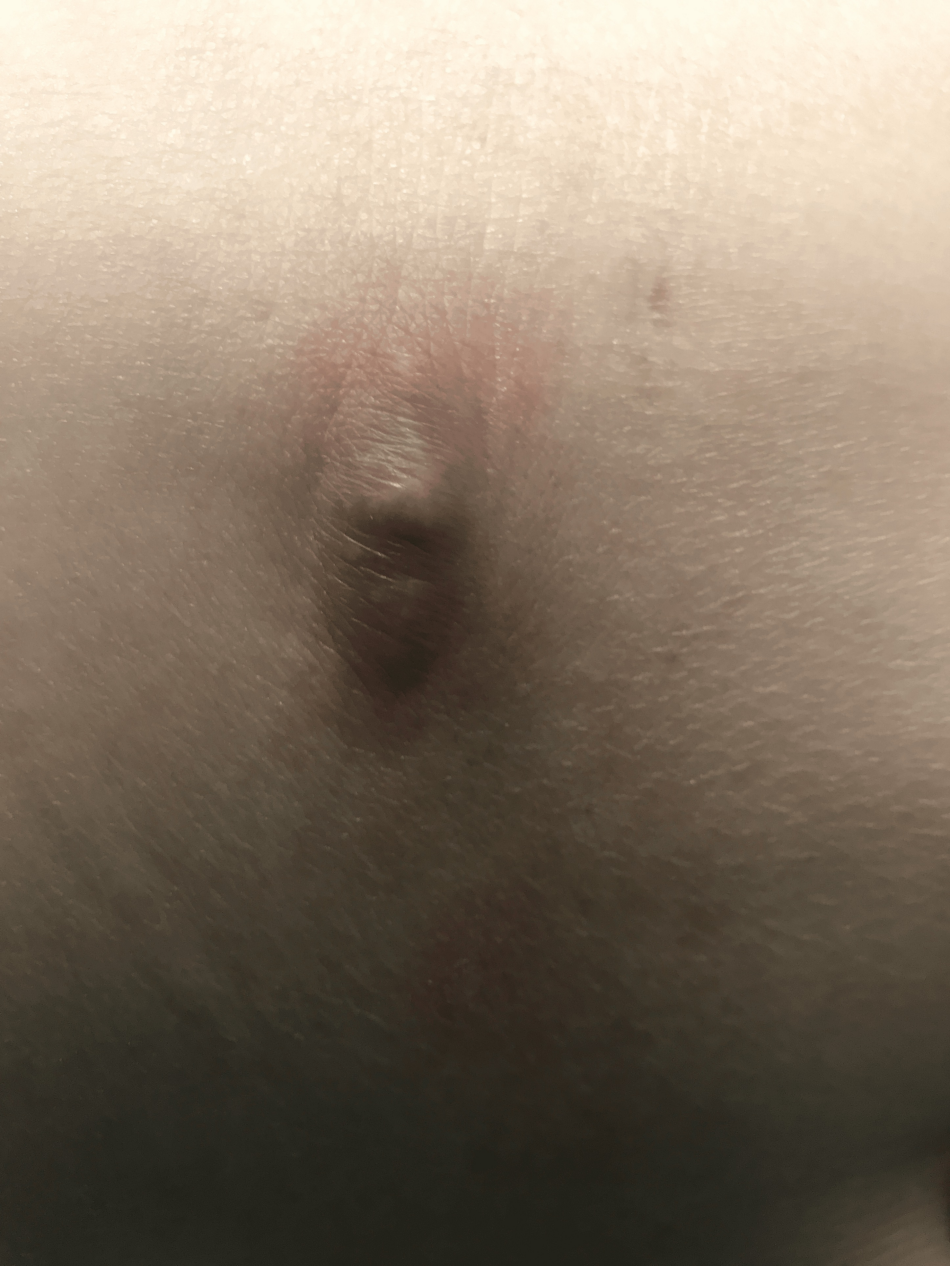
A 67-year-old woman with right dorso-lumbar pain two months after a fall from standing height, resulting in painful mass in the low back region of approximately 4 cm.

In April 2015, she sought consultation with his family physician due to right-sided lower back pain, that had started two months earlier after a fall from standing height, resulting in painful swelling of approximately 4 cm in the right dorso-lumbar region (Figure 1). After evaluation, her family doctor considered the possibility of a lipoma or post-traumatic hematoma. An ultrasound was requested, which revealed a large cystic formation measuring 12 mm, with calcifications inside (possibly a cortical cyst) on the periphery of the right kidney, and the patient was referred for a hospital urology consultation.

**Figure 2:**
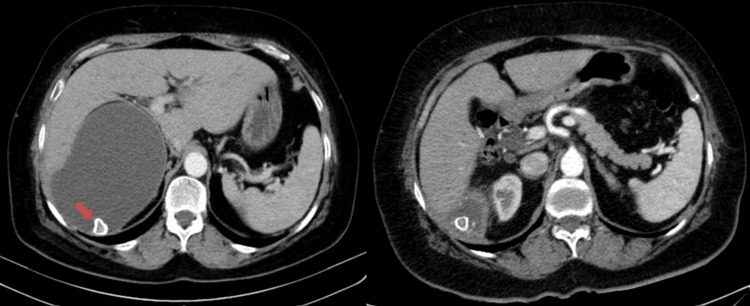
Axial CT image shows a foreign body (calculi) with calcified periphery (left, arrow) and a consequent reduction in the size of the fluid collection located in the hepatorenal space (right).

Subsequently, the patient developed inflammatory signs in the lumbar region, described with spontaneous drainage of stones, the analysis of which confirmed the composition of calcium oxalate. During urological follow-up, a computed tomography scan was performed, which revealed a significant reduction in the size of the fluid collection located in the hepatorenal space, with some gross calcifications inside it and a probable solution of continuity with the subcutaneous fat of the right lumbar region, although there is no evident subcutaneous organized fluid component in this imaging exam. The aforementioned collection in the hepatorenal space came into contact with the upper polar surface of the right kidney, as well as with the visceral face of the right hepatic lobe (Figure 2).

**Figure 3:**
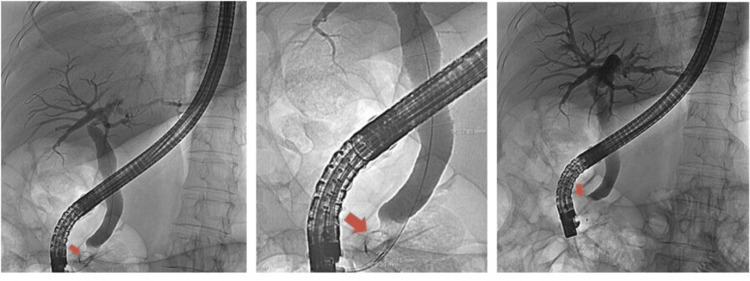
ERCP images show selective cannulation of the main bile duct, observing an image suggestive of a stone embedded in the papilla, measuring approximately 9 mm (arrow), with dilation of the intra and extrahepatic bile ducts (measuring 13 mm). Subsequent performance of sphincterotomy. ERCP: endoscopic retrograde cholangiopancreatography

At the hospital, after performing magnetic resonance cholangiopancreatography (MRCP), this condition was interpreted as a sequela of the previous cholecystectomy, having performed endoscopic retrograde cholangiopancreatography (ERCP), in which a 9-mm stone impacted in the papilla was removed, which caused dilation of the bile ducts (Figure 3). The patient remained asymptomatic for several months until new inflammatory signs appeared. She was seen by her family doctor and taken to the General Surgery Emergency Room, where local anesthesia was used to drain an abscess in that area, resulting in the release of a small amount of pus and numerous stones.

**Figure 4:**
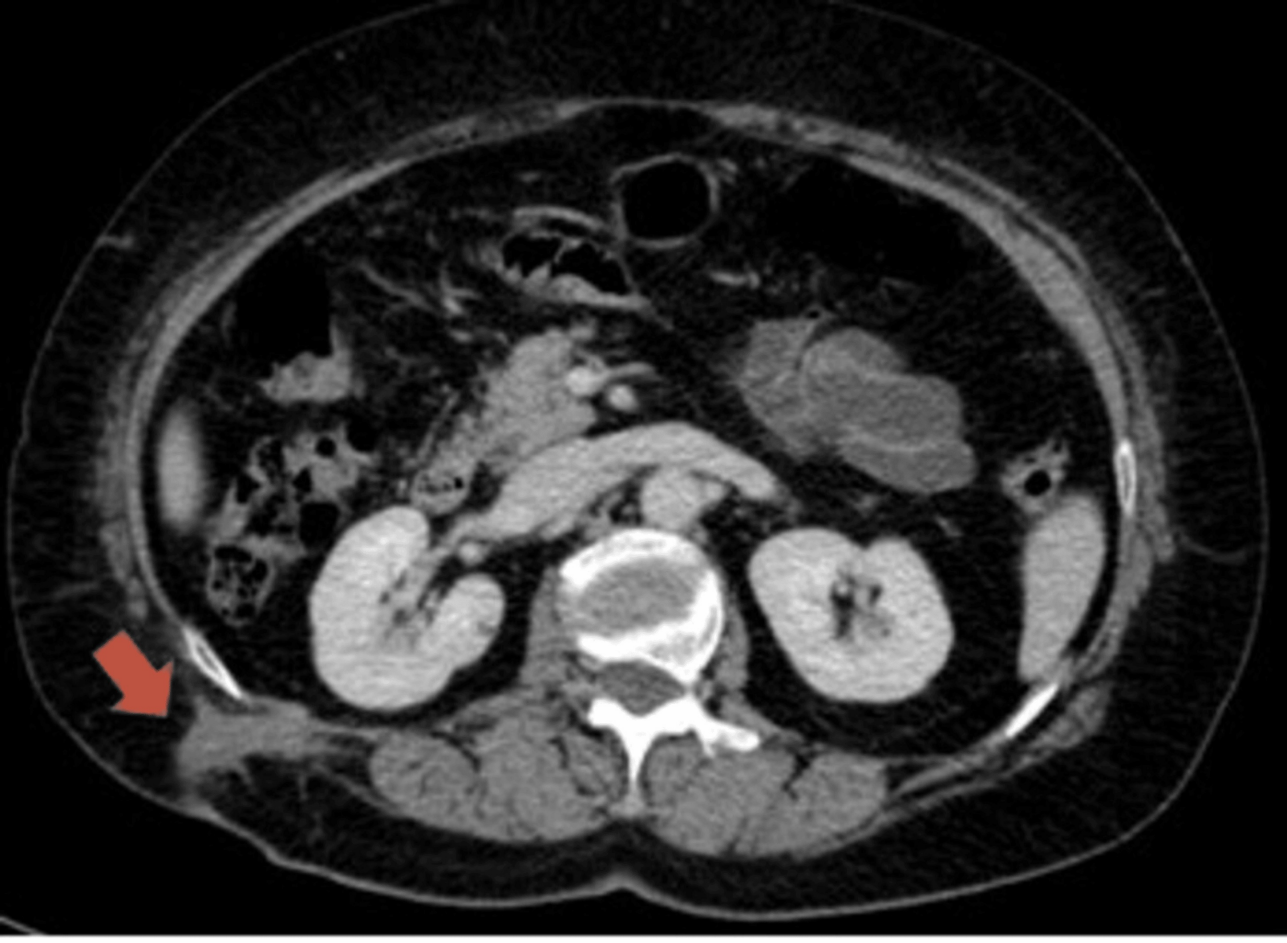
Axial CT image shows an 11-mm thick tubular densification extending to the hepatorenal space to the abdominal wall (arrow), within which small calcic foci were identified that drained spontaneously through the skin solution.

Subsequent CT images showed a small cavity containing several calculi, occupying the lateral aspect of the hepatorenal space and extending to the abdominal wall through an 11-mm thick tubular densification, within which small calcic foci were identified that drained spontaneously through the skin solution (Figure 4).

**Figure 5:**
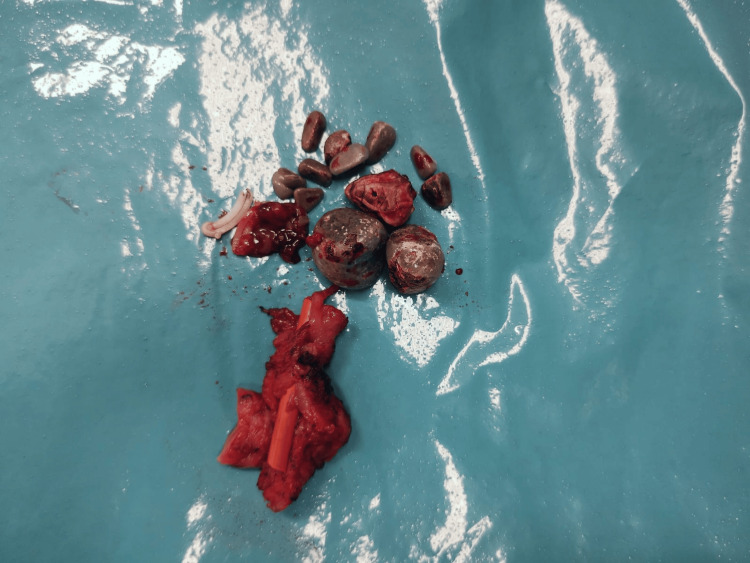
Biliary stones removed after partial fistulectomy and debridement of the fistulous tract.

The patient was proposed for surgery via a lumbar approach with partial fistulectomy and debridement of the fistulous tract for stone extraction (Figure 5). The patient showed favorable evolution without new episodes or pain complaints.

## Discussion

This clinical case illustrates a confluence of infrequent circumstances, such as the persistence of biliary stones following cholecystectomy and the development of a fistulous tract secondary to a traumatic hematoma [3,4]. Early reports on laparoscopic cholecystectomy stated that stones, left in the peritoneal cavity, had no deleterious effect. However, more recently, as a consequence of spilled stones, sepsis, adhesions and fistulae into abdominal organs or portal tracts have been described. Symptoms appeared from days to several months - fever, pain, and local or diffuse infection are common [4,5]. Some stones can shrink and even be reabsorbed (this explains why many patients remain asymptomatic) but some intra-abdominal stones will produce serous or purulent collections. Complications include abscesses and inflammatory masses which are generally confined to the subhepatic space or the retroperitoneum below the subhepatic space. This can result in bile duct injury, biliary leakage and infection with ectopic lithiasic drainage [6].

Beyond reporting a singular clinical event, this case emphasizes the crucial role of the family physician, who serves as a central figure in longitudinal care and in coordinating the differential diagnosis. A timely referral is often the key, as delayed diagnosis can lead to serious complications such as bile duct obstruction or secondary infections. So, it is necessary to identify these warning signs early, distinguishing them from other gastrointestinal disorders, and ensuring the patient undergoes appropriate imaging and specialist evaluation. Moreover, the ability to correlate the patient’s history and subtle symptoms allows the family doctor to act as a bridge between primary care and specialized treatment.

## Conclusions

This report highlights a rare post-cholecystectomy complication, simultaneously emphasizing the importance of the family physician's role in longitudinal follow-up and management of complex and unexpected clinical situations. In addition to early recognition and referral, a multidisciplinary approach is essential: gastroenterologists play a key role in confirming the diagnosis and managing bile duct stones, often performing ERCP for both diagnosis and treatment; radiologists contribute by interpreting imaging studies; general or hepatobiliary surgeons may be involved if surgical intervention is necessary. This multidisciplinary approach, involving close collaboration between primary and secondary healthcare services, was critical for the favorable clinical outcome. This case reinforces the importance of effective communication and coordination between different levels of healthcare to optimize the management and prognosis of complex clinical conditions.
